# Diversity and Bioactivity of Endophytic Actinobacteria Associated with Grapevines

**DOI:** 10.1007/s00284-022-03068-0

**Published:** 2022-11-03

**Authors:** Patanun Kanjanamaneesathian, Anish Shah, Hayley Ridgway, E. Eirian Jones

**Affiliations:** 1 Faculty of Agriculture and Life Sciences, Lincoln University, PO Box 85084, Lincoln, 7647 New Zealand; 2 The New Zealand Institute for Plant & Food Research Limited, Private Bag 4704, Christchurch, 8140 New Zealand

## Abstract

Grapevine trunk diseases (GTDs) are a significant problem for New Zealand viticulture. Endophytic actinobacteria are of interest as potential biocontrol agents due to their ability to inhibit plant pathogens and improve plant growth. However, no studies have investigated the diversity of actinobacteria associated with grapevines in New Zealand vineyards and their bioactivity. Actinobacteria diversity in different ‘Sauvignon blanc’ vine tissues from three vineyards (conventional and organic management, and different vine ages) was assessed using different methods and media. Forty-six endophytic actinobacteria were isolated, with more isolates recovered from roots (*n* = 45) than leaves (*n* = 1) and shoot internodes (*n* = 0). More isolates were recovered from the organic (*n* = 21) than conventional (*n* = 8) vineyard, mature (25-year old; *n* = 21) than young (2-year old; *n* = 2) vines and using a tissue maceration technique (*n* = 40). Actinomycete Isolation Agar, International Streptomyces Project 2, and Starch Casein media were effective for actinobacteria isolation. Most of the isolates recovered belonged to *Streptomyces*, with one isolate identified as *Mycolicibacterium*. Forty isolates were assessed for antifungal activity and plant growth-promoting (PGP) characteristics. Of these, 13 isolates had antifungal activity against test GTD pathogens (*Dactylonectria macrodidyma, Eutypa lata, Ilyonectria liriodendri, Neofusicoccum parvum*, and *N. luteum*)*.* Eighteen isolates exhibited more than one PGP trait; 25siderophore production (*n* = 25), phosphate solubilization (*n* = 6), and indole acetic acid production (*n* = 16). Two strains, *Streptomyces* sp. LUVPK-22 and *Streptomyces* sp. LUVPK-30, exhibited the best antifungal and PGP properties. This study revealed the diversity of culturable endophytic actinobacteria from grapevines in New Zealand vineyards and their biocontrol potential against GTD pathogens.

**Supplementary Information:**

The online version contains supplementary material available at 10.1007/s00284-022-03068-0.

## Introduction

The New Zealand viticulture industry is a significant contributor to the country’s economy, with export earnings in 2020 being approximately NZ$1.8 billion [1]. In 2021, the total wine production was estimated at more than 266 million L from 40,323 ha of production area. The main grape variety grown in New Zealand is ‘Sauvignon blanc’ accounting for 63% and 85% of the total grape planting and the nation’s exports, respectively [1]. However, grapevine trunk diseases (GTDs) are increasingly being recognized as having a major impact on the viticulture industry both in New Zealand and worldwide [2, 3]. In New Zealand, the incidence of GTDs was reported to be 20% in Marlborough and Hawke’s Bay vineyards [4]. Pruning wounds are the main point of entry for fungal spores, with the large number of wounds created both during the propagation process and in the vineyard increasing the risk of GTD infection [2]. The control strategies for GTDs are currently limited as there are no effective methods to eradicate the pathogens once they are established within the vines. Therefore, the focus is on prevention of GTDs using cultural practices to reduce the pathogen inoculum in the vineyard, such as remedial surgery by removing the visibly infected plant parts [5], and the application of fungicides to protect pruning wounds from infection [6]. However, chemicals can often only offer short-term protection. Moreover, due to the large amount of inoculum produced by the pathogens year-round and the year-round availability of susceptible wounds, frequent chemical applications are required to protect the vines from infection [2]. This results in a high input of chemicals to the vineyards, posing potential environmental and human health issues. For disinfection of grafted vines and propagation material, hot water treatment (HWT) has been reported to be an effective method [7]. However, researchers have reported that HWT is not effective at reducing all GTD pathogens under New Zealand propagation conditions [8, 9]. In addition, HWT can negatively impact the viability of cuttings and grafted material [8–10]. HWT has also been shown to increase the susceptibility of vines when subsequently challenged to the GTD pathogens *Neofusicoccum luteum* and *N. parvum*, potentially due to the impact of HWT on the microbial community associated with the vines [10]. Research into the development of biological control agents is a promising alternative strategy for the control of grapevine trunk pathogens.

Endophytes refer to microorganisms that colonize the internal plant tissue without causing any harm to the plant hosts [11]. This group of microbes can complete at least part of their life cycle in host tissues [12]. Strobel and Daisy [13] stated that more than 300,000 plant species have been identified as having one or more endophytes inhabiting their plant tissues. More importantly, some endophytes play a major role in promoting plant vigour in the presence of external environmental stresses such as, salinity, drought, soil nutrient deficiencies, and temperature or pH extremes, or diseases through the production of bioactive compounds, promoting plant growth, and/or induction of plant resistance [14].

Grapevine tissues harbour a variety of microbes, with endophytic actinobacteria being of particular interest due to their enormous diversity in terms of bioactivity [15]. In terms of their association with plants, endophytic actinobacteria are ubiquitous and are a source of novel bioactive compounds with diverse functions, including antimicrobials, plant growth promoters, and antioxidants [16]. There are a number of studies focussed on identifying the endophytic actinobacteria from various plants and their ability to produce bioactive compounds [17–19]. Other studies have reported that the actinobacterial community on grapevines is affected by tissue type [20, 21]. Further, different pest management strategies such as organic and integrated pest management (IPM) practices have also been reported to influence the diversity of endophytic actinobacteria in different grapevine cultivars [22]. However, there have only been a few studies of culturable endophytic actinobacteria carried out in New Zealand [18]. In addition, information regarding the diversity of culturable endophytic actinobacteria associated with grapevines under New Zealand vineyard conditions, and the influence of potential factors such as tissue type, age, and management practice on these communities is lacking. Since members of the endophytic actinobacterial communities are known to be able to promote plant growth and improve resilience to abiotic and biotic stresses, information regarding the endophytic actinobacterial communities associated with New Zealand grapevine plants is required. The aim of this research was to (i) identify the diversity of culturable endophytic actinobacteria associated with different grapevine tissues in New Zealand vineyards and determine the most effective method and media for their isolation; and (ii) investigate the potential biocontrol activity of actinobacteria isolates to inhibit key pathogens responsible for GTDs (*Dactylonectria macrodidyma, Eutypa lata*, *Ilyonectria liriodendri, Neofusicoccum parvum*, and *N. luteum*), and whether these endophytes produce bioactive compounds related to plant growth promotion traits using a range of in vitro bioactivity assays.

## Materials and Methods

### Grapevine Sampling Location and Sample Collection

Root, leaf, and shoot internode tissues were sampled from two 10-year-old *Vitis vinifera* ‘Sauvignon blanc’ clone 316 vines from both a conventionally managed block (CON) (− 43°38*′*47.3*"*S and 172°27*′*09.3*"*E) and an organically managed block (ORG) (43°38*′*59.5*"*S and 172°27*′*33.0*"*E) at the Lincoln University research vineyard, New Zealand in January 2021. The conventionally managed block is managed according to the Sustainable Winegrowing New Zealand program, whereas the organically managed block is managed according to the Organic Winegrowers New Zealand standards. Root and adjoining soil samples were collected using a soil corer from four directions (90° apart) around each plant approximately 10–15 cm from the stem base. One shoot from each side of the cordon was collected, with one fully-opened, apparently healthy, mature leaf sampled from the top, middle, and base of each shoot. Two shoot internode samples (~ 10 cm long) were excised from each selected shoot, the first shoot internode was sampled between the uppermost leaf and the next leaf down. The second shoot internode was sampled between the lowest leaf and the next leaf above. This resulted in one composite root/soil sample, six leaf samples, and four shoot internode samples from each vine. The plant samples were placed in separate clean plastic bags, transported back to the laboratory in a cold box and processed the same day.

Roots and shoot internodes were also collected from selected *Vitis vinifera* ‘Sauvignon blanc’ vines from the David Jackson’s experimental vineyard at Lincoln University in March 2021. This vineyard contains both mature 25-year-old vines (DJ25) (− 43.646619″S and 172.457664″E) and newly planted 2-year-old vines (DJ2) (− 43.646585″S and 172.457536″E), with the vines exposed to a similar climate and the same phytosanitary and fertilizer management. For both ages, one vine row in the middle of the relevant vineyard blocks was selected for the sampling. Six grapevine plants were randomly chosen from the selected row from each age using a random number generator (simple number generator app, Mac app store). Roots were collected as previously described. Shoot internodes were sampled from one green shoot and one woody shoot from 25-year-old vines, and two green shoots from 2-year-old vines, as previously described.

### Soil Analysis

The soil samples taken from each of the vineyard blocks were air-dried at room temperature for 3 days. Any large stones, plant tissue (roots or leaves), and insects/worms were removed using sterile forceps, and the soil samples were then sieved using a 2 mm stainless steel sieve, with the sieve cleaned and sterilized between each sample. Soil pH was determined using a 2:1 ratio of 30 mL deionized water and 15 g soil and the method described by Blakemore et al. [23]. Soil moisture content was determined by drying pre-weighed (20 g) soil samples in an oven at 105 °C for 24 h. After allowing the samples to cool down to room temperature in a desiccator, the samples were reweighed and used to determine the percentage soil moisture content for the soil samples [24]. The total N, soil organic matter, soil organic carbon, total carbon/nitrogen ratio and Olsen P of each soil sample were then determined by the Soil Analytical Laboratory, Lincoln University [24–27].

### Isolation of Endophytic Actinobacteria

The four soil sub-samples from each vine were used to make a composite sample. Root tissue fragments were removed using a sterile forceps with the fine (feeder) roots used for isolation. The remaining soil was placed in a sterile tube for further soil analysis.

All root, leaf, and shoot tissues were then washed with tap water to remove soil and other debris and air-dried in a sterile airflow in a laminar flow hood (Thermo Fisher Scientific, USA). The tissues were surface sterilized using a modification of the method described by Wicaksono et al. [28] by soaking in 70% ethanol for 30 s, followed by 2.5% sodium hypochlorite solution (NaOCl) amended with Tween 20 (1 mL per 1 L of solution) for 3 min for root and shoot samples, and 2 min for leaf samples. The tissue samples were then washed three times in sterile water for 1 min and dried in a laminar flow hood. A 0.2 mL aliquot of the last water wash was plated onto International Streptomyces Project 2 medium (ISP2; [29]) and incubated at 25 °C to check the effectiveness of the sterilization process. Lack of growth after 14 days incubation at 25 °C indicated that the surface sterilization process was effective.

Two different techniques were used to isolate actinobacteria from the grapevine tissue sampled for vines in the two different management blocks. Two replicate plates were set up for each tissue type/media for each of the techniques separately. All sterilized tissues were aseptically cut into small pieces (approximately 2 mm^2^). For the tissue plating technique [18], for each of the tissue sample, four tissue pieces were placed equidistant around the edge of two replicate plates for each of ISP2, Starch Casein (SC; [30]), Tap Water Yeast Extract agar (TWYE; [31]), and Actinomycete Isolation Agar (AIA; [32]). Each of the media was supplemented with cycloheximide (100 μg/mL) to inhibit fast-growing fungi [33, 34]. AIA and TWYE were also amended with nystatin (50 μg/mL) to inhibit fungi, and ISP2 and SC supplemented with nalidixic acid (30 μg/mL) to inhibit the growth of other bacteria [33, 34]. For the second technique, root samples were macerated using a modification of the methods described by Álvarez-Pérez et al. [34]. As this method did not successfully macerate leaf and green shoot internode tissue samples in a preliminary test, tissue maceration was only used for root samples in this study. A 0.1 g root sample was placed in separate 2 mL tubes containing 1.7 mL sterile water and approximately 15–20 sterile metal beads (2.0 mm diameter) and macerated using a FRITSCH homogenizer (John Morris Scientific Ltd) at 50 oscillations per second for 8 s, which was repeated 10 times per root sample with a 10 s rest between each time. A 0.1 mL aliquot of the macerated tissue samples was spread plated on two replicate plates of each of ISP2, SC, TWYE, and AIA using a sterile hockey stick. The plates were incubated at 25 °C for 7–14 days in the dark and observed for the development of colonies with morphology typical of actinobacteria, being powdery or elevated with margins pulling the agar.

For the grapevine tissues collected from different vine ages, root and shoot samples were processed and sterilized as previously described. However, since very few actinobacteria were recovered from shoot tissues in the previous sampling potentially due to the surface sterilization conditions being too harsh, the length of time in the 2.5% NaOCl solution was reduced to 2 min for woody shoots and 1 min for green shoots. Isolations from the green shoot tissue were only carried out using the tissue plating technique, with isolations from the root and woody stem tissue samples also carried out using the tissue maceration method as described. To improve the isolation of distinct individual colonies, the macerated tissue samples were further diluted tenfold for shoot tissue and 100-fold for root tissues. The tissue pieces and 0.1 mL aliquots of the diluted tissue macerates were plated on each of two plates of ISP2, SC, and AIA media.

### Identification of Actinobacteria

All 113 presumptive actinobacterial isolate colonies which grew on the plates were purified by subculturing onto fresh plates of the same medium (ISP2, SC, TWYE, and AIA). Each isolate was then subcultured onto fresh media and after 7 days growth at 25 °C in the dark, the macromorphological characteristics of the isolates including colony colour and characteristics, spore mass colour, colour of the substrate and aerial mycelium, and production of diffusible pigments were observed [33, 35]. Microscope slides were prepared using gram-staining methods [36] for one to two representative isolates from each of the morphological groups, to observe the structure of the mycelium, including the structure and appearance of the spores, under a light microscope (magnification 60x–100x). These characteristics were used to presumptively identify the selected isolates to genus level. All isolates were stored as liquid cultures at − 80 °C in 20% glycerol.

The isolates were identified based on sequencing of the *16S rRNA* gene. DNA of selected isolates was extracted using Extract-N-Amp™ Plant PCR Kit (Sigma-Aldrich) according to the manufacturer’s protocol. The DNA of the selected endophytic isolates was amplified using the actinobacteria specific primers F243 and R1494 [18]. The amplicons were then directly sequenced using the same primers as those used for amplification at the Lincoln University Sequencing Facility. In a further attempt to identify the endophytic actinobacteria isolates to species level, the *23S rRNA* and *rpoB* gene regions were sequenced for selected isolates. The *23SrRNA* gene region was amplified using primers F1067 and R2192 following the protocol outlined by Chaves et al. [37] and the *rpoB* gene region amplified using primers F2473 and R3303 as described by Adékambi et al. [38]. The amplicons obtained were purified using the QIAquick Gel Extraction Kit (QIAGEN Inc., Germany) and sequenced commercially by Macrogen Inc. (Seoul, South Korea) using the same primers used for the amplification.

The sequences obtained were corrected and the sequences assembled to create a consensus sequence using Geneious Prime® 2021.2.2 software (https://www.geneious.com). The sequences were compared with those of known origin using the Basic Local Alignment Search Tool (BLAST) in the GenBank database (http://www.ncbi.nlm.nih.gov), with 98.65% considered the cutoff value for species identification as proposed by Kim et al. [39]. For the *16S rRNA* gene region, the sequences were aligned using MUSCLE alignment aligned using the Muscle alignment tool (v 3.8.425 by Robert Edgar) in Geneious Prime® (v 2021.2.2), Biomatters Ltd. A phylogenetic tree was constructed using the Neighbour joining cluster analysis with Geneious Prime. Bootstrap analysis with 1000 re-samplings was used to evaluate the tree.

The sequences were deposited in NCBI with the accession numbers from OL347575, OL348273-OL348292, OL354988-OL354989 OL348293-OL348311 and OL354990-OL354993.

### In vitro Antifungal Activity

The bioactivity of actinobacteria isolates recovered from the grapevine tissue were tested in dual plate assays against five grapevine trunk pathogens, namely *D. macrodidyma* isolate LUPP1086*, Eutypa lata* isolate F70, *Ilyonectria liriodendri* isolate LUPP984*, Neofusicoccum parvum* isolate LUPP1507 and *N. luteum* isolate ICMP 16,678 obtained from the Lincoln University Plant Microbiology culture collection. A total of 40 actinobacteria isolates were used, as six isolates failed to regrow from the stored cultures. The actinobacteria isolates were streaked at one end of a Waksman agar (WA) [28] plate, 1 cm from the edge of the plate. For the actinobacteria isolates that had a confluence growth pattern, the cultures were inoculated at four equidistant points 1 cm from the edge of the WA plate using a sterile blunt-ended toothpick. The plates were then incubated at 25 °C for 7–10 days for the slow growing isolates and 2–4 days for the fast-growing isolates. After 7 days, a 6 mm diameter mycelial colonized agar plug taken from one of the fungal pathogens were then placed 5 cm from the actinobacteria colonies. Control plates were set up for each pathogen whereby a mycelial colonized agar disc was inoculated onto WA without the actinobacteria. Three replicate plates were set up for each treatment. All plates were incubated at 25 °C in a 12 h light and 12 h dark cycle for 7–14 days. Antifungal activity of the actinobacterial isolates was determined by measuring the inhibition zone between the actinobacteria and fungal colonies after 7 days for *N. luteum* and *N. parvum,* 12 days for *E. lata*, 14 days for *D. macrodidyma* and *I. liriodendri* using a digital calliper (Mitutoyo, Kanagawa, Japan). The antifungal activity of the isolates was classified based on the size of the inhibition zone against the test pathogens; high activity with inhibition zone > 5 mm (+ + +), moderate activity with inhibition zone < 5 mm but > 2 mm (+ +), low activity with inhibition zone < 2 mm but > 1 mm ( +), and no activity with no inhibition zone (−).

## Screening of Plant Growth-Promoting Traits

### Siderophore Production

The ability of the isolates to produce siderophores was tested using chrome azurol S–Luria Bertani (CAS-LB) agar as described by Dias et al. [40] which was modified from the CAS medium used by Schwyn and Neilands [41]. The actinobacterial strains were inoculated centrally onto the CAS-LB agar plates, with three replicate plates set up for each isolate and the uninoculated control. The plates were then incubated at 25 °C for 7–10 days. The width of any orange halo produced around the colony indicating the production of siderophore was recorded and compared with the uninoculated bacterial control plate. The width of the orange halo was measured from the edge of the colony in two perpendicular directions using a digital calliper. Siderophore production activity for each isolate was categorized into four classes based on the size of the zone around the colony; high activity with halo zone > 10 mm (+ + +), moderate activity with halo zone < 10 mm but > 5 mm (+ +), low activity with halo zone < 5 mm ( +), and no activity with no halo zone (−).

### Phosphate Solubilization

The ability of the endophytic actinobacteria to solubilize phosphate was determined on tricalcium phosphate (TCP) agar [42]. The actinobacteria culture was inoculated onto the centre of TCP agar plates and the plates were incubated at 25 °C in the dark for 7 days. Triplicate plates were set up for each isolate and the uninoculated control. The ability of the isolates to solubilize phosphate was determined by measuring the clear zone around the colony using a digital calliper. The presence of a clear zone was categorized into four classes based on the size of the zone around the colony; high activity with halo zone > 10 mm (+ + +), moderate activity with halo zone < 10 mm but > 5 mm (+ +), low activity with halo zone < 5 mm ( +), and no activity with no halo zone (−).

### Indole Acetic Acid (IAA) Production

IAA production was determined using the method described by Mishra et al. [43]. Sterile 1.7 mL tubes containing 1 mL of Luria Bertani broth amended with 5 mM of L-tryptophan (Sigma-Aldrich, New Zealand; LB + Trp) were inoculated with a loop full of an actinobacterial culture taken from a 7–10 day old culture. Three replicate tubes were set up for each isolate and the uninoculated control. After incubation at 25 °C in the dark for 7–10 days, the culture was centrifuged for 5 min at 10,000×*g* to remove bacterial cells. A 500 μL cell-free culture supernatant was then mixed with 500 μL of Salkowski reagent [44], and incubated at room temperature for 25 min to stabilize the colour change. The ability of the actinobacteria to produce IAA was then determined by visually comparing the pink colour intensity of the medium. A medium colour change from pale yellow to pink indicated the presence of IAA due to the conversion of L-tryptophan to IAA by the actinobacteria. The IAA production activity for each isolate was categorized into four classes depending on the intensity of the colour change; high activity with intense pink colour (+ + +), moderate activity with moderate pink colour (+ +), low activity with faint pink colour ( +), and no activity (−).

## Results

### Identification of Endophytic Actinobacteria

**Fig. 1:**
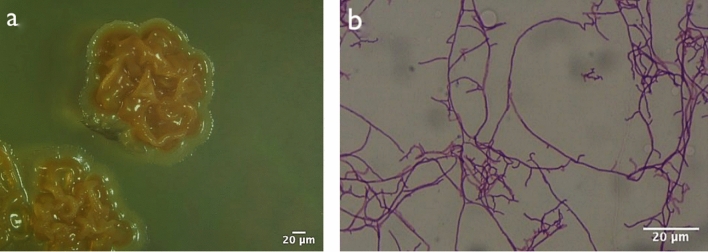
Characteristic appearance of **a** *Streptomyces* sp. isolate colonies observed under a stereomicroscope showing the characteristic colony morphology, and **b** mycelial characteristics from gram-stained slide preparation observed under a compound microscope (1000X) showing long filamentous, branched Gram positive structures

Based on the colony morphology and microscopic observations, 46 presumptive endophytic actinobacteria isolates were recovered from the grapevine tissues. All but one of the isolates were morphologically identified as members of the genus *Streptomyces* with long threadlike structures (Fig. 1A and B) and one isolate was classified as a member of the genus *Mycolicibacterium* with rod-shaped structure.

**Fig. 2:**
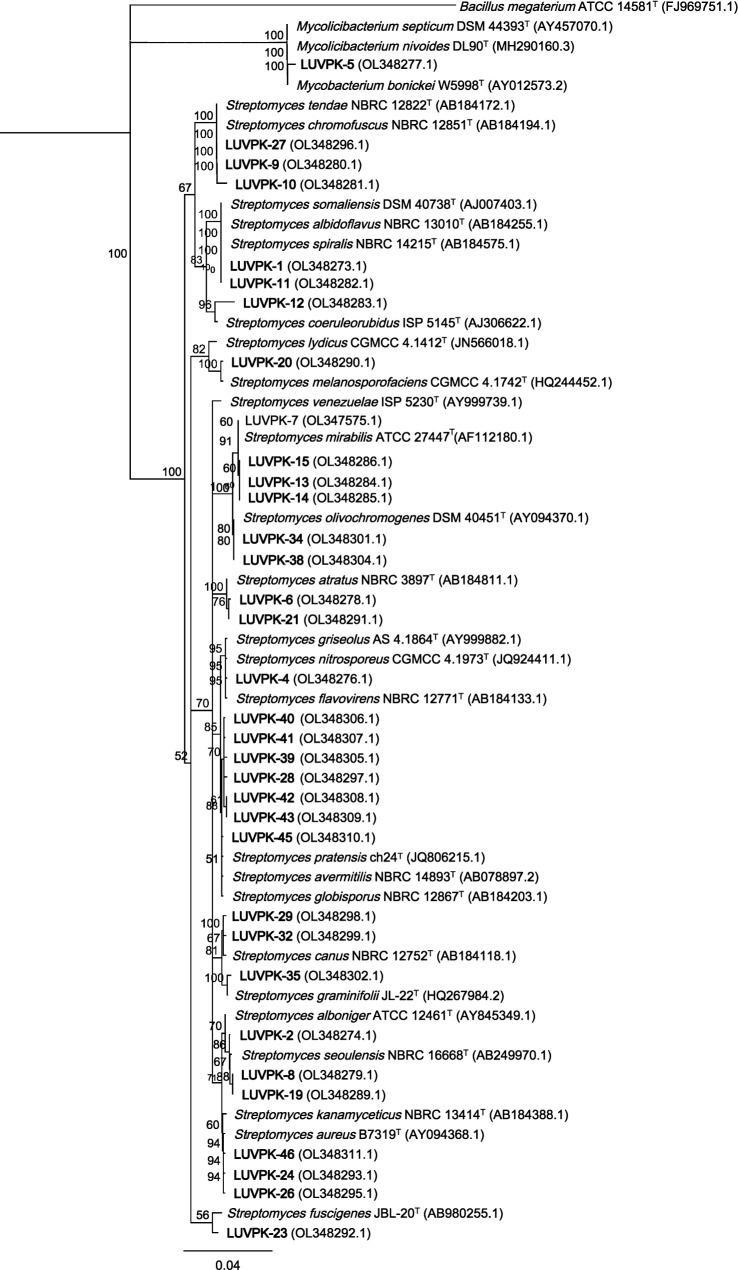
Neighbour joining phylogenetic tree based on an alignment of partial *16S rRNA* sequences of endophytic actinobacteria isolated from conventional, organic, old and young vines. The numbers at the branch nodes represent the bootstrap support value on 1000 re-samplings. The tree is rooted to the outgroup *Bacillus megaterium*

Analysis of the *16S rRNA* sequences indicated that the isolates recovered from the conventional (CON) and organic (ORG) vineyards were grouped into two families, *Streptomycetaceae* and *Mycobacteriaceae*. All but one of the 23 isolates, isolate LUVPK-5, were identified as belonging to the genus *Streptomyces* (*n* = 22) showing 96–100% similarity to sequences on GenBank (Fig. 2). Isolate LUVPK-5 was identified as belonging to the genus *Mycolicibacterium* with 99% identity. For the different vine ages (DJ2 and DJ25), all 23 isolates recovered were identified by *16S rRNA* gene sequences as belonging to the family *Streptomycetaceae*. For 12 isolates (LUVPK-3, LUVPK-16, LUVPK-17, LUVPK-18, LUVPK-22, LUVPK-25, LUVPK-30, LUVPK-31, LUVPK-33, LUVPK-36, LUVPK-37, and LUVPK-44), despite repeated attempts to amplify/sequence, only short sequences (< 500 bp) were obtained which could not be used to produce consensus sequences and therefore only the reverse sequences were used for BLAST analysis and these were not included in the phylogenetic tree (Fig. 2). The sequences were deposited in NCBI with the accession numbers OL347575, OL348273-OL348292, OL354988-OL354989, OL348293-OL348311, and OL354990-OL354993. The phylogenetic tree based on *16S rRNA* gene of endophytic actinobacteria grouped the sequences into two clades representing the genera *Streptomyces* and *Mycolicibacterium* along with sequences of type strains for species obtained from the GenBank database (Fig. 2). Some isolates could be identified to species such as LUVPK-7, LUVPK-13, LUVPK-14, and LUVPK-15 identified as *S. mirabilis*, isolates LUVPK-9, LUVPK-10, and LUVPK-27 grouped with a sequence from *S. chromofuscus* (AB184194.1), isolates LUVPK-34 and LUVPK-38 with *S. olivochromogenes* (AY094370.1), LUVPK-20 with *S. melanosporofaciens* (HQ244452.1), LUVPK-35 with *S. graminifolii* (HQ267984.2), and LUVPK-29 and LUVPK-32 with *S. canus* (Supplementary Table 1).

Some isolates could not be identified to species level based on sequences of the *16S rRNA* gene region. Isolates LUVPK-1 and LUVPK-11 were closely related to *S. spiralis* (AB184575.1), *S. albidoflavus* (AB184255.1), and *S. somaliensis* (AJ007403.1). Isolate LUVPK-5 was closely related to *M. septicum* (AY457070.1), *M. nivoides* (MH290160.3), and *M. boenickei* (AY012573.2). Isolate LUVPK-4 was aligned with *S. flavovirens* (AB184133.1), *S. nitrosporeus* (JQ924411.1), and *S. griseolus* (AY999882.1). Isolates LUVPK-28, LUVPK-39, LUVPK-40, LUVPK-41, LUVPK-42, LUVPK-43, and LUVPK-45 aligned with sequences of *S. pratensis* (JQ806215.1), S*. avermitilis* (AB078897.2), *S. globisporus* (AB184203.1). Isolates LUVPK-2, LUVPK-8, LUVPK-19, LUVPK24, LUVPK-26, and LUVPK-46 grouped together with *S. aureus* (AY094368.1), *S. kanamyceticus* (AB184388.1), and *S. alboniger* (AY845349.1) (Table 1, Fig. 2, Supplementary Table 1). The *23S rRNA* and/or *rpoB* gene regions for representative isolates for these were sequenced to attempt to confirm identity.

**Table 1:** BLAST analysis of *16S rRNA*, *23S rRNA*, and *rpoB* gene sequences from the closest type strains for endophytic actinobacteria isolates recovered from Sauvignon blanc grapevine tissues that could not be identified to species level from the partial *16S rRNA* gene sequences

Isolate	*16S rRNA* (%)	Accession no	*23S rRNA* (%)	Accession no	*rpoB* (%)	Accession no
LUVPK-1, 11	*Streptomyces spiralis* (99%)	AB184575.1	*S. antibioticus* (95.7%)	JF424141.1	*S. venezuelae* (91%)	FR845719.1#
	*S. albidoflavus* (99%)	AB184255.1			*S. lunaelactis* (89%)	KX503550.1
	*S. somaliensis* (99%)	AJ007403.1				
LUVPK-2, 8, 19	*S. aureus* (99%)	AY094368.1	*S. hiroshimensis* (76%)	JF424142.1	*S. avermitilis* (93%)	BA000030.4#
	*S. seoulensis* (99%)	AB249970.1			*S. lunaelactis* (91%)	KX503550.1
LUVPK-3*	*S. melanosporofaciens* (83%)	HQ244452.1	–	–	–	–
LUVPK-4	*S. flavovirens* (99.9%)	AB184133.1	*S. griseolus* (98%)	JQ806169	*S. avermitilis* (93%)	BA000030.4#
	*S. nitrosporeus* (99%)	JQ924411.1	*S. pratiensis* (97%)	JQ806204.1	*S. lunaelactis* (92%)	KX503550.1
	*S. griseolus* (98%)	AY999882.1				
LUVPK-9	*S. chromofuscus* (100%)	AB184194.1	*S. avermitilis* (96%)	NR_076331.1	*S. leeuwenhoekii* (95%)	LN831790.1#
					*S. lunaelactis* (89%)	KX503550.1
LUVPK-10	*S. chromofuscus* (100%)	AB184194.1	*S. avermitilis* (96%)	NR_076331.1	*S. leeuwenhoekii* (95%)	LN831790.1#
					*S. lunaelactis* (89%)	KX503550.1
LUVPK-27	*S. chromofuscus* (100%)	AB184194.1	*S. avermitilis* (96%)	NR_076331.1	*–*	–
LUVPK-16*	*S*. *coerulescens* (99.5%)	AJ399462	*S. avermitilis* (97%)	NR_076331.1	–	–
LUVPK-17*	*S. olivochromogenes* (97%)	AY094370.1	*S. antibioticus* (96%)	JF424141.1	–	–
LUVPK-18*	*S. mirabilis* (96%)	AF112180.1	*S. antibioticus* (96%)	JF424141.1	–	–
LUVPK-22*	*S. lydicus* (99%)	JN5660181.1	*S. antibioticus* (95%)	JF424140.1	*S. libani* (97%)	AP023408.1#
	*S. angustmyceticus* (96%)	AB184817			*S. lunaelactis* (92%)	KX503550.1
LUVPK-24, 26, 46	*S. aureus* (100%)	AY094368.1	*S. antibioticus* (95%)	JF424141.1	*S. avermitilis* (97%)	BA000030.4#
	*S. kanamyceticus* (99%)	AB184388.1			*S. lunaelactis* (90%)	KX503550.1
LUVPK-25*	*S. aureus* (99%)	AY094368.1	*S. antibioticus* (94%)	JF424141.1	*S. avermitilis* (93%)	BA000030.4#
					*S. lunaelactis* (90%)	KX503550.1
LUVPK-28	*S. pratensis* (98%)	JQ806215.1	*S. pratensis* (98%)	JQ806197	*S. venezuelae* (93%)	FR845719.1#
	*S. globisporus* (97%)	AB184203.1			*S. lunaelactis* (92%)	KX503550.1
LUVPK-30*	*S. lasalocidi* (99%)	MK852399	*S. antibioticus* (97%)	JF424141.1	*S. avermitilis* (97%)	BA000030.4#
					*S. lunaelactis* (89%)	KX503550.1
LUVPK-31*	*S. mirabilis* (98%)	AF112180.1	*S. antibioticus* (97%)	JF424141.1	*S. avermitilis* (98%)	BA000030.4#
					*S. lunaelactis* (91%)	KX503550.1
LUVPK-33*	*S. mirabilis* (98%)	AF112180.1	*S. antibioticus* (97%)	JF424141.1	*S. avermitilis* (98%)	BA000030.4#
					*S. lunaelactis* (90%)	KX503550.1
LUVPK-34, 38	*S. olivochromogenes* (99%)	AY094370.1	*S. antibioticus* (96%)	JF424141.1	*S. avermitilis* (97%)	BA000030.4#
	*S. mirabilis* (98%)	AF112180.1			*S. lunaelactis* (90%)	KX503550.1
LUVPK-37*	*S. olivochromogenes* (97%)	AY094370.1	*S. antibioticus* (97%)	JF424141.1	*S. avermitilis* (98%)	BA000030.4#
					*S. lunaelactis* (90%)	KX503550.1
LUVPK-36*	*S. fagopyri* (98%)	MN044908	*S. antibioticus* (97%)	JF424141.1	*–*	*–*
	*S. mirabilis* (98%)	AF112180.1				
LUVPK-39	*S. pratensis* (99%)	JQ806215.1	*S. pratensis* (98%)	JQ806197	*S. venezuelae* (92%)	FR845719.1#
					*S. lunaelactis* (92%)	KX503550.1
LUVPK-40	*S. pratensis* (99%)	JQ806215.1	*–*	*–*	*S. venezuelae* (93%)	FR845719.1#
					*S. lunaelactis* (92%)	KX503550.1
LUVPK-41	*S. pratensis* (99%)	JQ806215.1	*S. pratensis* (99%)	JQ806197	*S. venezuelae* (93%)	FR845719.1#
					*S. lunaelactis* (92%)	KX503550.1
LUVPK-42	*S. pratensis* (99%)	JQ806215.1	*S. pratensis* (99%)	JQ806197	*S. venezuelae* (93%)	FR845719.1#
					*S. lunaelactis* (92%)	KX503550.1
LUVPK-43	*S. pratensis* (99%)	JQ806215.1	*–*	*–*	*–*	*–*
LUVPK-45	*S. pratensis* (99%)	JQ806215.1	*–*	*–*	*–*	–
LUVPK-44*	*S. pratensis* (98%)	JQ806215.1	*S. pratensis* (98%)	JQ806197	*–*	–
	*S. microflavus* (97%)	AB184434				

*Indicates isolates where the forward and reverse 16SrRNA sequences were not able to be used to produce a consensus sequence with the BLAST results based on one direction (forward or reverse)

#Complete genome sequence

Of the 12 isolates (LUVPK-3, LUVPK-16, LUVPK-17, LUVPK-18, LUVPK-22, LUVPK-25, LUVPK-30, LUVPK-31, LUVPK-33, LUVPK-36, LUVPK-37, and LUVPK-44) which only resulted in short sequences (< 500 bp) for *16S rRNA* gene, all but isolate LUVPK-3 were successfully amplified using primers for the *23S rRNA* and/or *rpoB* gene regions and produced high quality sequences (Table 1).

Of the 46 isolates, the *23S rRNA* gene region was successfully amplified for 29 isolates yielding products of approximately 1000 bp, whilst for the *rpoB* gene region, 24 isolates were successfully amplified with the products being 700–1000 bp. The analysis of the sequences by BLASTN confirmed that all isolates belonged to the genus *Streptomycetes.* The *23S rRNA* sequences were deposited in NCBI with the accession numbers ON076430-ON0677458 and the *rpoB* sequences with the accession numbers ON260877-ON260900. Based on the *23S rRNA* gene sequences isolate LUVPK-16 was identified as being closely related to *Streptomyces* sp. (NR_076331.1), isolates LUVPK-17 and LUVPK-18 closely related to *Streptomyces* sp. (JF424141.1) (96% similarity) (Table 1). Sequences of the *23S rRNA* and *rpoB* gene regions identified isolate LUVPK-22 as *Streptomyces sp.* JF424140.1 and AP023408.1 with 95% and 97% similarity. Isolates LUVPK-30, LUVPK-31, LUVPK-33, LUVPK-36, and LUVPK-37 were identified as *Streptomyces* sp. based on sequences of the *23S rRNA* gene region and LUVPK-31 and LUVPK-33 and LUVPK-30 as *S. avermitilis* for the *rpoB* sequences with 97–98% similarity. Isolate LUVPK-44 was closely related to *S. pratensis* with 98% similarity from *23S rRNA* sequence.

### Isolation and Relative Abundance of Grapevine Associated-Actinobacteria

#### Effect of Management Practice

A total of 23 endophytic actinobacteria isolates were recovered from sterilized tissues of *Vitis vinifera* ‘Sauvignon blanc’ clone 316. Of these, isolates were more frequently recovered from root tissue (*n* = 22), and only one isolate was recovered from leaf from the bottom of the selected shoot. No isolates were recovered from either the leaf from the top of the selected shoot or the middle leaf. For the different vineyard management practices, a higher frequency of endophytic actinobacteria isolates was recovered from vines in the organically managed (*n* = 15, 65.22%) compared with the conventionally managed (*n* = 8, 34.78%) vineyard sites. The one *Mycolicibacterium* sp. isolate was recovered from root tissue from the conventionally managed vineyard, and for *Streptomyces* spp., seven isolates were recovered from the conventionally managed vineyard and 15 isolates from the organically managed vineyard. The one *Streptomyces* sp. isolate recovered from leaf tissue was recovered from the conventionally managed vineyard.

Two isolation methods were compared to recover endophytic actinobacteria from grapevine tissues: (i) direct plating of tissue on the selective media and (ii) plating homogenized root tissues on the selective media. For method (i) the putative actinobacteria colonies were selected and subcultured onto fresh media. For method (ii), the representative colonies that were characteristic of actinobacteria were subcultured onto fresh media to get the pure colonies. The tissue plating method resulted in the recovery of six isolates (26.1%) with 17 isolates recovered using the tissue maceration method (73.9%). The one *Mycolicibacterium* sp. isolate was recovered from tissue plating method. According to the management factors, 2 and 6 isolates found in conventional vineyard were recovered by tissue plating (25.0%) and maceration techniques (75.0%), respectively. From the organically managed vineyard, four isolates (26.6%) were recovered using tissue plating, and 11 isolates (73.3%) using maceration technique. The time taken for actinobacteria colonies to grow from the tissues onto the agars differed for the various selective agars. Colonies appeared after 3–4 days incubation on TWYE compared with 7–10 days on AIA and SC, with colonies appearing 12–14 days on ISP2 agar. Eight isolates were recovered on AIA (34.8%), seven isolates on ISP2 (30.4%), five isolates on SC (21.7%) and three isolates were obtained on TWYE agar (13.0%). The one *Mycolicibacterium* sp. isolate was recovered on AIA, with *Streptomyces* spp. isolates recovered on all four media.

The *16S rRNA* phylogenetic analysis revealed the relationship of isolate group clusters and their isolation sources. *Streptomyces* sp. isolates obtained from both the conventionally and organically managed vineyards were clustered in the same group (Fig. 2). However isolate LUVPK-4, recovered from the conventionally managed vineyard grouped separately from those recovered from the organically managed vineyard. The phylogeny analysis also indicated that the different media were not selective as to the species recovered. For example, isolates that were closely matched to *S. atratus* were recovered on both AIA and SC agar, *S. aureus* recovered from AIA, SC and TWYE, and *S. mirabilis* recovered on both ISP2 and TWYE plates.

#### Effect of Different Vine Ages

All 23 endophytic actinobacteria isolates were recovered from sterilized root tissues of *Vitis vinifera* ‘Sauvignon blanc’ using the tissue maceration method. None were recovered from either green or woody shoot tissues. Twenty-one isolates were recovered from the mature vines (DJ25, 89.3%) compared with only two isolates from newly planted vines (DJ2, 10.7%). The tissue plating method was used only for the green shoot tissue and tissue maceration method was used for the root and woody shoot samples. Of the three selective media used, 17 isolates were recovered on SC (73.9%), five isolates on AIA (21.7%), and one isolate on ISP2 (4.3%). Of the two isolates recovered from young vines, LUVPK-35 grouped with isolates recovered from those obtained from older vines, whilst LUVPK-27 did not. The effect of isolation agar showed that isolates that were closely related to *S. aureus* were obtained on both ISP2 and SC plates, whilst those closely related to *S. pratensis* were recovered on both AIA and SC.

### Soil Physicochemical Analysis

The soil moisture content, pH, and nutrient contents (C, N, total C/N, and Olsen P) were determined for the soils collected from the four vineyard sites (Table 2). More actinobacteria were recovered from the grapevine roots from the organically managed vineyard site which had a lower soil moisture level (3.84%), compared with the conventionally managed vineyard site which had a higher soil moisture level (10.01%). The soil from the organically managed site also had a higher pH (6.07) and lower Olsen P (18 µg/g) compared with the conventionally managed site (pH of 5.87 and Olsen P of 58 µg/g). The values for the other soil nutrients including percentage carbon (C), percentage nitrogen (N), and Total C/N ratio were similar between the two vineyard sites. A higher number of actinobacteria were recovered from the grapevine roots from the mature 25-year-old vines which had a lower soil moisture level (18.7%) compare with newly planted 2-year-old vines which had a higher soil moisture level (22.5%). The soil from the mature 25-year-old vine site also had a higher pH (6.18) and lower Olsen P (20.0 µg/g). The values for the other soil nutrients including percentage carbon (%C), percentage nitrogen (%N), and total C/N ratio were similar between the two vineyard sites.

**Table 2:** The percentage soil moisture, pH, and carbon (%), nitrogen (%) and Olsen P (µg/g) of soil samples collected from the organic (ORG), conventionally (CON) managed, old (DJ25), and young (DJ2) vineyard sites at the Lincoln University vineyard

Soil factors	Vineyard sites			
	Conventional (CON)	Organic (ORG)	Old (DJ25)	Young (DJ2)
% Soil moisture	10.01	3.84	18.69	22.47
pH	5.87	6.07	6.18	5.7
% C	2.35	2.21	1.67	3.24
% N	0.20	0.19	0.16	0.17
Total C/N	11.7	11.4	10.3	19.0
Olsen P (µg/g)	58.00	18.00	20.0	195.0

### In vitro Antifungal Activity

**Fig. 3:**
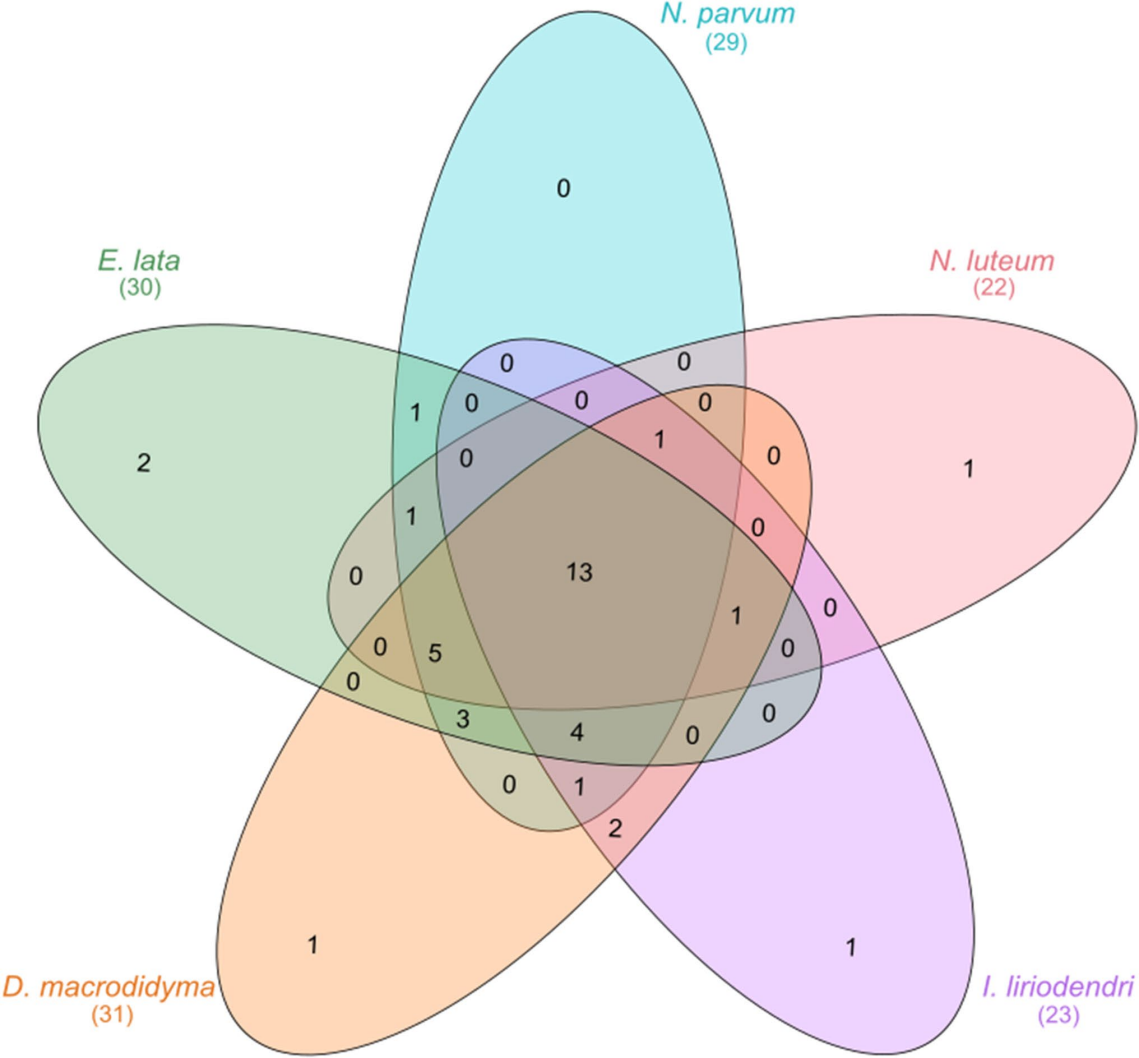
Venn diagram illustrating the number of endophytic actinobacteria isolates showing high and moderate inhibitory activity against five fungal trunk pathogens (*Dactylonectria macrodidyma*, *Eutypa lata*, *Ilyonectria liriodendri*, *Neofusicoccum parvum*, and *N. luteum*) in the dual culture assay. Total number of isolates with high and moderate inhibitory activity for each pathogen indicated in the parenthesis

The inhibitory activity of 40 endophytic actinobacteria isolates including 7 isolates from the conventionally managed (CON), 11 from organically managed (ORG), two from newly planted (DJ2), and 20 from mature (DJ30) vines were determined. A total of 13 endophytic actinobacteria showed either high or moderate antifungal activity against all five of the phytopathogenic fungi responsible for grapevine trunk diseases (Fig. 3). All of these isolates were recovered from roots, with 8 isolates recovered from mature vines, 1 from a young vine, 7 from the organic vineyard site and 2 from the conventional vineyard site (Supplementary Table 2). Five endophytic actinobacteria isolates suppressed the growth of only one pathogen, two isolates only inhibited the growth of *E. lata* and one isolate each only had inhibitory activity against *N. luteum*, *I. liriodendri*, and *D. macrodidyma.* Only three isolates had no activity against any of the fungal pathogens. Across all of the actinobacterial isolates, *N. parvum* was the most susceptible pathogen with a mean inhibition zone of 1.51 cm, followed by *D. macrodidyma* and *E. lata* with mean inhibition zones of 0.93 cm and 0.91 cm, respectively. The mean inhibition zone across all actinobacterial isolates for *N. luteum* was 0.39 cm and *I. liriodendri* was the most resistant with a mean inhibition zone of 0.28 cm.

### Plant Growth-Promoting Traits

Of the 40 endophytic actinobacteria isolates screened for plant growth-promoting traits 18 isolates (45%) exhibited more than one plant growth-promoting trait. Three isolates had high or moderate activity for two of the plant growth-promoting traits performed (isolates LUVPK-22, LUVPK-30, and LUVPK-34).

For siderophore production 25 isolates (62.5%) were positive for siderophore production on CAS-LB plates, four isolates showed high siderophore activity, with two isolates recovered from mature vines and one isolate recovered from both the conventional and organic vineyard sites. Three isolates showed moderate activity with two isolates recovered from the mature vines and one isolate from the organic site. Most of the endophytic actinobacteria tested had low siderophore activity with 15 isolates negative for siderophore production (Supplementary Table 2).

Most actinobacteria were negative for phosphate solubilization, whilst six isolates (15%) were shown to solubilize phosphate on TCP plates. Two isolates showed moderate activity with a clear zone of ≥ 2 mm with both isolates recovered from grapevine roots, one each from the conventional and organic vineyard sites (Supplementary Table 2). Four isolates showed low phosphate solubilizing activity (clear zone < 1 mm).

Sixteen of the assessed endophytic actinobacteria isolates were indicated to be able to produce IAA, 15 isolates were recovered from root tissues and one isolate from leaf tissue. Eight of the isolates were recovered from mature vines, two of the isolates from young vines, four isolates from the organic and two isolates from the conventionally managed vineyard sites (Supplementary Table 2). Most of the isolates (*n* = 13) showed low IAA production, with only three isolates producing an intense pink colour indicating strong IAA production, with these all recovered from mature vines.

Of the actinobacterial isolates screening, one isolate LUVPK-22, identified as closely related to *Streptomyces lydicus* (99% similarity) had the highest antifungal activity against all grapevine trunk pathogens and moderate siderophore production and phosphate solubilization activity (Supplementary Table 2).

## Discussion

This is the first study to identify the diversity of culturable endophytic actinobacteria associated with *Vitis vinifera* ‘Sauvignon blanc’ tissues in New Zealand and to investigate the potential bioactive repertoire of these isolates in terms of inhibition of grapevine trunk pathogens and plant growth promotion traits. All but one of the isolates recovered were identified as belonging to the genus *Streptomyces,* with the only other genus recovered being *Mycolicibacterium*. This is similar to the other studies where *Streptomyces* was the most commonly isolated actinobacterial genus from grapevine tissues [34] and other woody plant hosts [18, 45, 46]. However, in contrast with the results of the current study these reported that comparatively higher diversity of other actinobacteria genera were recovered from grapevine tissues. For example, Álvarez-Pérez et al. [34] reported that although 45% of the 58 endophytic actinobacteria isolated from grapevine root tissue were identified as belonging to the genus *Streptomyces*, isolates of six other genera were also recovered. In the only other study of endophytic actinobacteria carried out in New Zealand, Purushotham et al. [18] reported that of the nine actinobacterial isolates recovered from the New Zealand medicinal plant *Pseudowintera colorata* (Horopito), *Streptomyces* along with *Micromonospora* were the most commonly isolated genera, with one isolate each of *Norcardia*, *Microlunatus*, and *Nakamurella* also recovered. The reason for the lack of recovery of other genera in this study is unclear. This study employed similar isolation techniques and selective media used in other studies [33, 38, 48] so this is unlikely to be the reason for failure to isolate more diverse genera. In the current study actinobacteria colonies were recovered into pure culture after 14 days incubation, in contrast much longer incubation periods of up to 4 weeks were used by other researchers [34, 47, 48], with Kaewkla and Franco [45] incubating the isolation plates for up to 16 weeks. This may have resulted in the recovery of slower growing genera.

In this study, the majority of the endophytic actinobacteria were recovered from the root tissues compared with the above ground tissues sampled, leaf and stem tissue. This result is consistent with previous studies both on grapevines and other plant hosts [20, 49]. This indicated that the root tissue of grapevine represents the richest source of endophytic actinobacteria. Actinobacteria are common members of the soil microbial community [50], and are a likely source of endophytes colonizing the root tissue.

Both isolation methods used were successful in recovering endophytic actinobacteria from the grapevine root tissues. The tissue maceration method resulted in a higher recovery of actinobacteria colonies which is likely due to the cell disruption process facilitating the release of the endophytes from the plant tissues [51]. However, this method did not result in recovery of actinobacteria from the stem tissue. Future work is recommended to optimize both the cell disruption period and the dilution level for each tissue type to improve recovery [51]. Since the majority of the actinobacteria isolates recovered belonged to the genus *Streptomyces* modification of the media by supplementing with amino acids such as L-asparagine, arginine, and proline, which have been reported to increase the recovery of different actinobacteria species, may also result in more actinobacterial genera being recovered [33, 51, 52].

Although sequencing of the *16S rRNA* confirmed that the majority of the isolates belonged to the genus *Streptomyces* (*n* = 45), it was not sufficient to resolve the identity of a number of the isolates to species level. Additional sequencing of *23rRNA* and *rpoB* gene regions did not provide any further improved taxonomic resolution for most isolates. This was due to these either not being discriminatory between closely related species or as sequences for these gene regions are not available in the databases for the type strains for many species. The lack of ability to identify the strains to species level means that it was not possible to determine whether any of the factors investigated influenced the actinobacterial species recovered, or to identify the potential antifungal metabolites produced based on taxonomy. Álvarez-Pérez et al. [34] also reported that multi locus sequence analysis with housekeeping genes, *atpD* (ATP synthase F1, beta subunit), *gyrB* (DNA gyrase B subunit), *recA* (recombinase A), *rpoB* (RNA polymerase, beta subunit), and *trpB* (tryptophan synthase, beta subunit) were not able to identify all the *Streptomyces* strains recovered in their study to species level. Whole genome sequencing is increasingly been used to analyse the taxonomy of bacteria, including actinobacteria [53] and could be use in the future to further determine the identity of the strains endophytically colonizing grapevines.

Twenty-five-year-old vines were colonized by more actinobacteria (*n* = 21), with only two *Streptomyces* isolates recovered from 2-year-old vines. This suggested that plant maturity plays an important role in shaping the grapevine endomicrobiome, probably due to the increased time available for colonization, changes in root exudates affecting rhizosphere-microbe interactions, and tissue development stage [54, 55]. However, other reports have indicated that endophytic actinobacteria were more frequently recovered from the stems of younger vines [56] or that there was no significant difference between ages [57]. Culture independent methods, such as Illumina-based next generation sequencing approaches, should be used to determine the complete communities associated with grapevines, and to confirm whether *Streptomyces* is the dominant genus endophytically colonizing vines.

Management practice also influenced the relative abundance of endophytic actinobacteria. More isolates were recovered from the organically managed vineyard compared to the conventional vineyard. This suggested that external inputs such as, cultural practices and the application of fertilizer and pesticides could influence microbial communities [22]. In the current study the majority of the actinobacteria isolates were recovered from the root tissues and these endophytes are likely to represent a sub-population of the rhizosphere microbiome [58], therefore any factors that affect these soil communities are likely to affect the recruitment of endophytes by the roots. Organic management has been suggested to increase the population and diversity of soil microbial communities which may enhance endophytic colonization of the roots [59]. In addition to the difference in practices, vines in the conventionally managed site had been confirmed to be free of grapevine leaf roll viruses, whilst the organically managed vines were much lower in overall health and likely to be virus infected, and this may have also affected the endophytic actinobacterial communities. In the current study, the three actinobacterial isolates which had high activity against all five GTD pathogens were isolated from the organically managed low health vines. However, since none of the vines sampled were symptomatic for GTD further work should investigate the impact of different vine health status, especially related to GTD on these endophytic communities with the aim to identify species or strains associated with high health vines that might have activity to protect vines from pathogens [60].

In addition to antifungal activity, a large number of the actinobacteria isolates were positive for PGP traits with 45% of isolates exhibiting more than one trait. Specifically, 62.5% of isolates were positive for siderophore production on CAS-LB, 15% of isolates shown to solubilize phosphate on TCP plates and 40% of isolates indicated to produce IAA. However, since bacteria can produce other molecules such as indole pyruvic acid, and indoleacetamid which can also react with the Salkowski reagent to result in a pink colour [61], confirmation of IAA production by these actinobacterial isolates using HPLC is required. The results showed that overall, the PCP traits assayed for in this study were widespread in the endophytic actinobacteria associated with grapevines being similar to other studies for actinobacteria recovered from grapevine rhizosphere soil [47], as endophytes of *Camellia* spp. [46], *P. colorata* [18] and *Rhynchotoechum ellipticum* [33]. Further, the studies of Purushotham et al. [18] and Borah et al. [46] reported inoculation of plants with selected endophytic actinobacterial strains promoted plant growth. This study identified two strains, *Streptomyces* sp. LUVPK-22 and *Streptomyces* sp. LUVPK-30, that exhibited the best antifungal and PGP properties and these warrant further investigation to determine their effect for both disease control and promoting growth of vines.

The majority of the soil physiochemical factors assessed were similar for the different vineyard sites, and likely not to have influenced the actinobacterial communities recovered from the roots. However, more endophytic actinobacteria isolates were recovered from vineyard site with the lower soil moisture, suggesting that these endophytes are more resistant to dryer soil conditions [62] due partly to their ability to form resistant exospores.

Endophytic actinobacterial isolates inhibited at least one of the phytopathogens*,* with many isolates inhibiting the growth of several of these pathogens. Other studies have reported the antifungal effects of actinobacteria recovered from grapevines and associated soils or other plants against a range of grapevine pathogens [18, 34, 47]. Some of the tested isolates were positive for one or more trait associated with promoting plant growth but had limited activity against pathogens. This suggests that these beneficial traits are not linked. Further, the *Streptomyces* sp. strains that were positive for the different beneficial traits were distributed across the diversity of the species recovered. This indicates that there is no clear association between activity and species identity for the members of the genus *Streptomyces,* with bioactivity appearing to be a strain rather than species characteristic. However, as discussed, further work is required to confirm the species identity of the isolates recovered in the study.

## Conclusion

This study provided the first information of the diversity of culturable endophytic actinobacteria diversity associated with grapevine in New Zealand vineyards. Members of the genus *Streptomyces* were identified as the dominant members of the grapevine endophytic actinobacterial communities, with most of the isolates recovered from the root tissue. The present study demonstrated that culture-dependent methods were able to isolate actinobacterial isolates, although dominated by members of the genus *Streptomyces*. Integration of culture-independent approach such metabarcoding will facilitate more information on grapevine-associated actinobacterial communities. The current study also extended the information on the bioactivity of the endophytic actinobacterial strains, in terms of biocontrol activity against GTD associated pathogens and plant growth-promoting traits. *Streptomyces* spp. LUVPK-22 and LUVPK-30 were identified as the most promising candidates for future study to determine their biocontrol activity *in planta.*

## Supplementary Information

Below is the link to the electronic supplementary material.

Supplementary file1 (DOCX 65 KB)

## Data Availability

Sequences were deposited in GenBank with the Accession Numbers OL347575, OL348273-OL348292, OL354988-OL354989 OL348293-OL348311 and OL354990-OL354993, ON076430-ON076458 and ON260877-ON260900.
