# Supplementary material for: Diversity and Bioactivity of Endophytic Actinobacteria Associated with Grapevines
Source: Curr Microbiol. 2022 Nov 3;79(12):390. doi: 10.1007/s00284-022-03068-0 (PMC9633489; doi:10.1007/s00284-022-03068-0)
Supplement: Supplementary file 1 — Supplementary file1 (DOCX 65 KB) [file 284_2022_3068_MOESM1_ESM.docx]

Supplementary Table 1. Identification of endophytic actinobacteria isolates according to selective media, isolation techniques and vineyards.

| Isolate no. | 16S rRNA Closest species | Type strain Accession no. | Similarity | Isolate identified as | Media name | Isolation technique | Vineyard / tissue |
| --- | --- | --- | --- | --- | --- | --- | --- |
| LUVPK-1, 11 | *Streptomyces spiralis*  *S. albidoflavus*  *S. somaliensis* | AB184575.1  AB184255.1  AJ007403.1 | 99%  99%  99% | *Streptomyces* sp. | AIA  AIA | Tissue Plate  Tissue Plate | ORG / Root  ORG / Root |
| LUVPK-2 | *S. aureus*  *S. seoulensis*  *S. kanamyceticus* | AY094368.1  AB249970.1  AB184388.1 | 99%  99%  96% | *Streptomyces* sp. | TWYE | Maceration | HH / Root |
| LUVPK-3 | *Streptomyces melanosporofaciens* | HQ244452.1 | 83% | *Streptomyces* sp. | AIA | Maceration | HH / Root |
| LUVPK-4 | *S. flavovirens*  *S. griseolus*  *S. nitrosporeus* | AB184133.1  AY999882.1  JQ924411.1 | 99.91%  98%  99% | *Streptomyces* sp. | AIA | Tissue Plate | HH / Leaf |
| LUVPK-5 | *Mycolicibacterium septicum*  *M. nivoides*  *M. boenickei* | AY457070.1  AY012573.2  NR_029036.1 | 99.31%  99%  99% | *Mycolicibacterium* sp. | AIA | Maceration | HH / Root |
| LUVPK-6, 21 | *S. atratus*  *S. atratus* | AB184811.1 | 99%  99% | *Streptomyces atratus* | AIA  SC | Maceration  Tissue Plate | ORG / Root  ORG / Root |
| LUVPK-7, LUVPK-13, LUVPK-14, LUVPK-15, LUVPK-18 | *S. mirabilis*  *S. mirabilis*  *S. mirabilis*  *S. mirabilis*  *S. mirabilis* | AF112180.1 | 100%  100%  99%  100%  96% | *Streptomyces mirabilis*  *Streptomyces mirabilis*  *Streptomyces mirabilis*  *Streptomyces mirabilis*  *Streptomyces* sp. | ISP2  ISP2  TWYE  TWYE  ISP2 | Maceration  Maceration  Maceration  Maceration  Maceration | HH / Root  ORG / Root  ORG / Root  ORG / Root  ORG / Root |
| LUVPK-8 | *S. aureus* | AY094368.1 | 100% | *Streptomyces aureus* | AIA | Maceration | ORG / Root |
| LUVPK-9,10,27 | *S. chromofuscus*  *S. tendae* | AB184194.1  AB184172.1 | 99%  96% | *Streptomyces* sp. | AIA | Maceration  Maceration  Maceration | ORG / Root  ORG / Root  DJ2 / Root |

Supplementary Table 1 Continued

| Isolate no. | 16S rRNA Closest species | Type strains Accession no. | Similarity | | Isolate identified as | Media name | Isolation technique | Vineyard / tissue |
| --- | --- | --- | --- | --- | --- | --- | --- | --- |
| LUVPK-12 | *S. coeruleorubidus* | AJ306622.1 | 99.9% | | *Streptomyces coeruleorubidus* | SC | Tissue Plate | HH / Root |
| LUVPK-16 | *S*. *coerulescens*  *S. venezuelae* | AJ399462  AY999739.1 | 99.5%  98% | | *Streptomyces* sp. | ISP2 | Maceration | HH / Root |
| LUVPK-17 | *S. olivochromogenes*  *S. mirabilis* | AY094370.1  AF112180.1 | 96.61%  98% | | *Streptomyces* sp. | SC | Maceration | ORG / Root |
| LUVPK-19 | *S. aureus* | AY094368.1 | 100% | | *Streptomyces aureus* | SC | Tissue Plate | ORG / Root |
| LUVPK-20 | *S. melanosporofaciens* | HQ244452.1 | | 99.75% | *Streptomyces melanosporofaciens* | SC | Maceration | ORG / Root |
| LUVPK-22* | *S. angustmyceticus*  *S. lydicus* | AB184817  JN566018.1 | | 96%  99% | *Streptomyces lydicus* | SC | Tissue Plate | ORG / Root |
| LUVPK-23 | *S. fuscigenes* | AB980255.1 | | 99.16% | *Streptomyces fuscigenes* | ISP2 | Maceration | ORG / Root |
| LUVPK-24, 26, 46 | *S. aureus*  *S. alboniger*  *S. kanamyceticus* | AY094368.1  AY845349.1  AB184388.1 | | 100%  96%  96% | *Streptomyces* sp. | SC  SC  ISP2 | Maceration  Maceration  Maceration | DJ25 / Root  DJ25 / Root  DJ25 / Root |
| LUVPK-25 | *S. aureus*  *S. alboniger*  *S. kanamyceticus* | AY094368.1  AY845349.1  AB184388.1 | | 99%  96%  97% | *Streptomyces aureus* | SC | Maceration | DJ25 / Root |
| LUVPK-28 | *S. pratensis*  *S. globisporus* | JQ806215.1  AB184203.1 | | 98%  97% | *Streptomyces* sp. | SC | Maceration | DJ25 / Root |
| LUVPK-29, 32 | *S. canus* | AB184118.1 | | 99% | *Streptomyces canus* | SC  SC | Maceration  Maceration | DJ25 / Root  DJ25 / Root |
| LUVPK-30 | *S. lasalocidi*  *S. mirabilis* | MK852399  AF112180.1 | | 99%  97% | *Streptomyces* sp. | SC | Maceration | DJ25 / Root |
| LUVPK-31, 33 | *S. mirabilis*  *S. avermitilis* | AF112180.1  AB078897.2 | | 98%  98% | *Streptomyces* sp. | SC  SC | Maceration  Maceration | DJ25 / Root  DJ25 / Root |

Supplementary Table 1 Continued

| Isolate no. | 16S rRNA Closest species | Type strains Accession no. | Similarity | Isolate identified as | Media name | Isolation technique | Vineyard / tissue |
| --- | --- | --- | --- | --- | --- | --- | --- |
| LUVPK-34, 37, 38 | *S. olivochromogenes*  *S. mirabilis*  *S. avermitilis* | AY094370.1  AF112180.1  AB078897.2 | 99%  98%  98% | *Streptomyces* sp. | SC  SC SC | Maceration  Maceration  Maceration | DJ25 / Root  DJ25 / Root  DJ25 / Root |
| LUVPK-35 | *S. graminifolii* | HQ267984.2 | 99% | *Streptomyces graminifolli* | SC | Maceration | DJ2 / Root |
| LUVPK-36 | *S. fagopyri*  *S. mirabilis* | MN044908  AF112180.1 | 98%  98% | *Streptomyces* sp. | SC | Maceration | DJ25 / Root |
| LUVPK-44 | *S. microflavus*  *S. pratensis* | AB184434  JQ806215.1 | 97%  98% | *Streptomyces* sp. | AIA | Maceration | DJ25 / Root |
| LUVPK-39, 40, 41, 42, 43, 45 | *S. pratensis*  *S. globisporus* | JQ806215.1  AB184203.1 | 99%  96-97% | *Streptomyces* sp. | SC  SC  SC  AIA  AIA  AIA | Maceration  Maceration  Maceration  Maceration  Maceration  Maceration | DJ25 / Root  DJ25 / Root  DJ25 / Root  DJ25 / Root  DJ25 / Root  DJ25 / Root |

Supplementary Table 2. Activity of endophytic actinobacteria isolates against five fungal trunk pathogens (*Eutypa lata (El)*, *Neofusicoccum parvum (Np),* *N. luteum (Nl), Ilyonectria liriodendri (Ii)* and *Dactylonectria macrodidyma (Dm)*, siderophore production on chrome azurol S (CAS-LB) agar, phosphate solubilization on tricalcium phosphate agar (TCP), and indoleacetic acid (IAA) production on Luria Bertani broth amended with L-tryptophan (LB+Trp).

| Isolate | Tissue/ Site | *El*^a^ | *Np*^a^ | *Nl*^a^ | *Ii*^a^ | *Dm*^a^ | TCP^b^ | LB+Trp^c^ | CAS-LB^d^ |
| --- | --- | --- | --- | --- | --- | --- | --- | --- | --- |
| LUVPK-1 | Root / ORG | +++ | +++ | +++ | +++ | +++ | - | **-** | + |
| LUVPK-2 | Root / CON | ++ | +++ | +++ | + | +++ | - | ++/+ | + |
| LUVPK-4 | Leaf / CON | +++ | +++ | ++ | - | +++ | - | +/- | + |
| LUVPK-5 | Root / CON | +++ | +++ | +++ | ++ | +++ | - | - | + |
| LUVPK-6 | Root / ORG | +++ | +++ | +++ | ++ | +++ | - | +/- | - |
| LUVPK-7 | Root / CON | +++ | +++ | +++ | ++ | +++ | - | - | - |
| LUVPK-9 | Root / ORG | +++ | +++ | +++ | ++ | +++ | - | - | - |
| LUVPK-11 | Root / ORG | +++ | +++ | +++ | +++ | +++ | - | - | - |
| LUVPK-12 | Root / CON | +++ | - | +++ | +++ | +++ | - | - | + |
| LUVPK-13 | Root / ORG | +++ | +++ | + | +++ | +++ | - | - | - |
| LUVPK-15 | Root / ORG | +++ | +++ | ++ | +++ | +++ | - | +/- | - |
| LUVPK-16 | Root / CON | - | - | - | - | +++ | ++ | - | + |
| LUVPK-17 | Root / ORG | +++ | +++ | ++ | ++ | +++ | - | +/- | + |
| LUVPK-19 | Root / ORG | - | - | - | ++ | + | + | - | - |
| LUVPK-20 | Root / CON | + | + | - | ++ | +++ | + | - | +++ |
| LUVPK-21 | Root / ORG | - | + | - | +++ | +++ | + | - | +++ |
| LUVPK-22 | Root / ORG | +++ | +++ | +++ | +++ | +++ | ++ | - | ++ |
| LUVPK-23 | Root / ORG | ++ | +++ | + | - | - | - | + | + |
| LUVPK-25 | Root / OLD | +++ | +++ | + | +++ | +++ | + | +/- | + |
| LUVPK-26 | Root / OLD | +++ | +++ | ++ | ++ | +++ | - | ++ | + |
| LUVPK-27 | Root / YOUNG | - | +++ | - | +++ | +++ | - | +/- | + |

Supplementary Table 2. Continued

| Isolate | Tissue/ Site | *El*^a^ | *Np*^a^ | *Nl*^a^ | *Ii*^a^ | *Dm*^a^ | TCP^b^ | LB+Trp^c^ | CAS-LB^d^ |
| --- | --- | --- | --- | --- | --- | --- | --- | --- | --- |
| LUVPK-28 | Root / OLD | + | +++ | ++ | ++ | +++ | - | - | - |
| LUVPK-29 | Root / OLD | - | - | - | - | - | - | - | - |
| LUVPK-30 | Root / OLD | +++ | +++ | +++ | ++ | +++ | - | ++ | ++ |
| LUVPK-31 | Root / OLD | +++ | +++ | +++ | + | +++ | - | - | - |
| LUVPK-32 | Root / OLD | ++ | +++ | ++ | ++ | +++ | - | - | - |
| LUVPK-33 | Root / OLD | +++ | +++ | +++ | + | +++ | - | +/- | +++ |
| LUVPK-34 | Root / OLD | ++ | +++ | - | ++ | +++ | - | ++ | +++ |
| LUVPK-35 | Root / YOUNG | +++ | +++ | +++ | ++ | +++ | - | + | + |
| LUVPK-36 | Root / OLD | +++ | +++ | +++ | ++ | +++ | - | - | - |
| LUVPK-37 | Root / OLD | +++ | +++ | +++ | - | - | - | +/- | ++ |
| LUVPK-38 | Root / OLD | +++ | +++ | + | + | +++ | - | - | - |
| LUVPK-39 | Root / OLD | +++ | +++ | + | ++ | +++ | - | - | + |
| LUVPK-40 | Root / OLD | +++ | +++ | +++ | ++ | +++ | - | - | + |
| LUVPK-41 | Root / OLD | ++ | +++ | + | ++ | +++ | - | - | + |
| LUVPK-42 | Root / OLD | ++ | ++ | +++ | + | +++ | - | +/- | + |
| LUVPK-43 | Root / OLD | +++ | +++ | ++ | ++ | +++ | - | - | + |
| LUVPK-44 | Root / OLD | +++ | +++ | ++ | ++ | +++ | - | - | + |
| LUVPK-45 | Root / OLD | +++ | +++ | - | + | +++ | - | - | + |
| LUVPK-46 | Root / OLD | +++ | +++ | ++ | +++ | +++ | - | +/- | - |

CON- Conventionally managed vineyard, ORG- Organically managed vineyard sampled in Jan-Feb 2021

DJ2- Newly planted vines (2-year-old), and DJ25- Mature vines (25-year-old) sampled in Feb-March 2021

^a^Dual culture assay on Waksman plates: (+++): high activity (inhibition zone > 5 mm), (++): moderate activity (inhibition zone < 5 mm but ≥ 2 mm) and (+): low activity (inhibition zone < 2 mm but > 1 mm).

^b^Siderophore production on CAS-LB plates: (+++): high activity (halo zone ≥ 10 mm), (++): moderate (halo zone 10 mm < x > 5 mm), (+): low (clear zone < 5 mm), and (-): no halo zone observed.

^c^Phosphate solubilization on TCP plates: (+++): high activity (halo zone ≥ 10 mm), (++): moderate (halo zone 10 mm < x > 5 mm), (+): low (clear zone < 5 mm), and (-): no halo zone observed.

^d^IAA production on LB+Trp culture: (+++): intense pink colour, (++): moderate pink colour, (+): faint pink colour, and (-): no color change observed.
